# Association of 25-hydroxy vitamin D level with the blood pressure response to a maximum exercise test among professional indoor athletes

**DOI:** 10.1007/s00421-020-04421-6

**Published:** 2020-06-25

**Authors:** Pascal Bauer, Lutz Kraushaar, Oliver Dörr, Timm Bauer, Holger Nef, Christian W. Hamm, Astrid Most

**Affiliations:** 1 Department of Cardiology and Angiology, Justus- Liebig- University Giessen, Giessen, Germany; 2 Adiphea GmbH, Werbach, Germany; 3 Department of Cardiology and Intensive Care Medicine, Sana Clinic Offenbach, Offenbach, Germany; 4 Department of Cardiology, Kerckhoff Clinic GmbH, Bad Nauheim, Germany

**Keywords:** Exercise test, Professional athletes, Indoor sports, Performance, Vitamin D, Blood pressure response, Hypertension

## Abstract

**Purpose:**

Low vitamin D levels have been associated with elevated blood pressure (BP) in the general population. However, whether there is an association of vitamin D insufficiency with BP changes during maximum exercise in athletes is currently unclear.

**Methods:**

A total of 120 male professional indoor athletes (age 26 ± 5 years) were examined. BP was measured at rest and during a graded cycling test. We assessed the BP response (BPR) during maximum exercise and the respective load. BP and BPR (peak-baseline BP) were analysed with respect to 25-OH vitamin D levels, with levels < 30 ng/mL defining vitamin D insufficiency.

**Results:**

35 athletes were classified as being vitamin D insufficient. BP was not different between sufficient and insufficient vitamin D groups (122 ± 10/75 ± 7 vs. 120 ± 12/77 ± 9 mmHg). At maximum exercise, however, systolic BP (198 ± 17 vs. 189 ± 19, *p* = 0.026) and the pulse pressure (118 ± 18 vs. 109 ± 21 mmHg, *p* = 0.021) were higher in the sufficient group; the BPR was not different between groups (76 ± 20/5 ± 6 vs. 69 ± 22/3 ± 6 mmHg, *p* = 0.103). Athletes with sufficient levels had a higher maximum power output (3.99 ± 0.82 vs. 3.58 ± 0.78 W/kg, *p* = 0.015) and achieved higher workloads (367 ± 78 vs. 333 ± 80 W, *p* = 0.003). The workload-adjusted BPR (maximum systolic BP/MPO) was not different between athletes with sufficient and insufficient vitamin D levels (51 ± 10 vs. 56 ± 14 mmHg × kg/W, *p* = 0.079).

**Conclusion:**

Athletes with sufficient vitamin D achieved a higher maximum systolic BP and a higher maximum power output. The workload-adjusted BPR was not different between groups, which suggests that this finding reflects a better performance of athletes with sufficient vitamin D.

## Introduction

Insufficiency and deficiency of 25-OH vitamin D have been shown to be associated with arterial hypertension (Kunutsor et al. 2013; Wang 2016), increased cardiovascular events, impaired vascular function (Al Mheid et al. 2011), and even cardiovascular mortality (Wang 2016; Anderson et al. 2010; Vimaleswaran et al. 2014). The fact that vitamin D receptors are found throughout the cardiovascular system, in particular in smooth muscle cells (Somjen et al. 2005), myocytes, and endothelial cells (Norman 2008), suggests an impact of vitamin D on cardiovascular function. Further, it was found that supplementation of vitamin D in asymptomatic deficient subjects leads to improved endothelial function and evokes a relaxation of smooth muscle cells (Tarcin et al. 2009). Vitamin D regulated reduction of the activity of the renin–angiotensin–aldosterone system also bespeaks a crucial role of vitamin D in the cardiovascular system (Forman et al. 2010). Taken together, all of this evidence points to mechanisms that potentially mediate a blood pressure-regulating effect of vitamin D.

Healthy individuals with normal blood pressure (BP) at rest but an exaggerated BP response (BPR) during exercise are at higher risk of developing arterial hypertension (Lewis et al. 2008; Holmqvist et al. 2012). In addition, a recent meta-analysis revealed that exercise-induced systolic BP ≥ 196 mmHg predicted cardiovascular events with a sensitivity of 62% and a specificity of 75% (Percuku et al. 2019). The clinical impact of BP response during exercise, however, remains a controversial issue (Kim and Ha 2016; Schultz et al. 2015), and upper reference values for the general population are not well established (Schultz et al. 2013; Gupta et al. 2007; Weiss et al. 2010; Currie et al. 2018). The European Society of Cardiology states in its latest guideline that there is currently no consensus on normal BPR during exercise (Williams et al. 2018). In previous guidelines, a systolic peak BP of 210 mmHg for men and 190 mmHg for women were proposed as diagnostic thresholds for the general population (Mancia et al. 2013). Recommendations for athletes are absent in both guidelines, yet recently published studies proposed different thresholds for defining an exaggerated BPR to exercise (Caselli et al. 2019; Pressler et al. 2018). A higher exercise-induced BP response in athletes was explained as representing a superior exercise performance compared with that of the general population (Caselli et al. 2016). Thus far, there is no consensus about “normal” and “exaggerated” BPR in athletes. In addition, the clinical relevance of “exaggerated” exercise-induced BP in athletes is currently unclear, although a higher risk for development of arterial hypertension in these athletes was suggested (Caselli et al. 2019). In competitive triathletes, an exaggerated BPR to exercise testing was associated with a higher prevalence of myocardial fibrosis (Tahir et al. 2018), raising concerns about potential arrhythmic consequences, including sudden cardiac death (Zorzi et al. 2016).

To date, only a few studies have addressed the BPR to maximum exercise in elite athletes (Pressler et al. 2018; Caselli et al. 2019), and none of these studies reported 25-OH vitamin D status. Vitamin D insufficiency and deficiency is commonly found in athletic populations (Owens et al. 2015; He et al. 2016; Mehran et al. 2016; Maroon et al. 2015). Our group recently discovered a high prevalence of vitamin D insufficiency (25-OH vitamin D < 30 ng/mL) in professional handball athletes in Germany (Bauer et al. 2018). Further, we recently demonstrated that vitamin D-insufficient elite handball athletes displayed a significantly higher peripheral and central BP compared with the values in athletes with sufficient vitamin D levels (Bauer et al. 2019).

Given the acknowledged physiological influences of vitamin D on endothelial and smooth muscle cells (Somjen et al. 2005), the haemodynamic effects of vitamin D insufficiency might be amplified during exercise. As regular exercise, performed at moderate intensity, is a well-known therapeutic tool for lowering BP (Williams et al. 2018), it is obvious that controlling for both vitamin D and cardiorespiratory fitness status is necessary to gain reliable insights about the impact of vitamin D on BP at rest and the BPR to exercise.

Therefore, this study was undertaken to evaluate the association between 25-OH vitamin D levels, brachial BP at rest, and the BPR to a maximum exercise test in elite indoor athletes. Both handball and ice hockey, with their many interval sprints, are team sports that expose athletes to a high haemodynamic stress. Both were classified as a sport with a high dynamic component (> 75% VO2max) and a moderate static component (10–20%) (Levine et al. 2015). In addition, both are indoor sports with a higher risk of vitamin D insufficiency compared to outdoor sports (Krzywanski et al. 2016).

## Materials and methods

The study was carried out at the university hospital in Giessen, Germany, which is located near 50° N latitude. It was conducted as a cross-sectional study of professional athletes during the routine pre-season medical monitoring program of the first German handball division and the second German ice hockey division. Data were collected in the second half of July in the years 2015–2018. Athletes were studied during the summer, when 25-OH vitamin D levels are expected to have reached their peak (Morton et al. 2012; Krzywanski et al. 2016).

The following criteria for serum 25-OH vitamin D concentrations were chosen according to recently published studies and recommendations (Holick et al. 2011, 2012; Pludowski et al. 2013; Priemel et al. 2010): values < 30 ng/mL were defined as insufficient and values ≥ 30 ng/mL were defined as sufficient 25-OH vitamin D levels.

The examination took place at noon between 12:00 and 14:00 o’clock and was scheduled in the 1st week of the new season after a 6-week competition-free interval. The last time athletes had trained was 36 h prior to the study beginning; the last meal was breakfast approximately 3 h before the investigation. There was no restriction of caffeine intake provided. Thus, alcohol consumption was prohibited the two days prior to the study beginning. The day before the examination was filled with commercial dates without physical effort.

### Study population

The participants were 120 healthy, injury-free professional handball and ice hockey athletes of varying nationalities. All athletes included were Caucasians with white skin and none of them was a regular sunbed user. None took vitamin D supplements or other multivitamin supplements. All individuals were subjected to a physical examination, 12-lead electrocardiogram (ECG), cardiopulmonary exercise test, and blood testing. Age, height, weight, body mass index, serum 25-OH vitamin D, calcium, magnesium and parathyroid hormone levels were determined. Body surface area was calculated using the formula of DuBois (Du Bois and Du Bois 1989). Maximum workload, maximum power output, heart rate at rest, brachial BP at rest, maximum heart rate, and maximum BP were assessed. Players then were divided into groups according to vitamin D levels as described above, and statistical analyses were performed.

### Laboratory testing

Blood samples were drawn from an antecubital vein in a sitting position. Blood samples for plasma analyses were collected into two 7.5 mL S-Monovette^®^ tubes (Sarstedt AG & Co. KG, Germany), one containing lithium heparin. An additional 2.7 mL sample, with dipotassium ethylenediamine tetra-acetic acid (K2EDTA) as anticoagulant, was acquired (Sarstedt AG & Co. KG, Germany). Automated analysis was carried out within 30 min of blood draw. Serum 25-OH vitamin D concentrations were determined with a Liaison diagnostic system (DiaSorin, Stillwater, MN, USA) by chemiluminescent immunoassay. The range of detection is 4–150 ng/mL with a precision of 5.0% CV and an accuracy SD of 1.2. Parathyroid hormone was analysed using an electrochemiluminescent immunoassay (Elecsys PTH (1–84)^®^, Roche Diagnostics, Germany), which measures the circulating active parathyroid hormone. The range of detection is 5.5–2300 pg/mL with a precision range of 2.5–3.4% CV. Furthermore, calcium levels, a complete blood cell count, and a basic metabolic panel including electrolytes were assessed and analysed by a Modular Analytics E 170 module (Roche Diagnostics, Mannheim, Germany).

All participants received a clear explanation of the study and provided their written informed consent. The local ethics committee of the University of Giessen approved the study protocol. The study meets current ethical standards (Harriss et al. 2017).

### Blood pressure measurements at rest before the exercise testing

Resting brachial BP was measured before the exercise testing using a validated automatic device based on a standard sphygmomanometer technique (Boso clinicus, Bosch + Sohn GmbH & Co. KG, Germany). The cuff used for measurement was adjusted to the individual’s arm circumference. Measurements were performed by a trained research associate on both arms in a sitting position after a resting period of 5 min and repeated after 2 min. The average BP for each arm was calculated and the highest value was used for statistical analyses. Athletes with a resting BP > 140 mmHg systolic or > 90 mmHg diastolic were excluded from the study.

### Exercise testing and assessment of maximum blood pressure

Athletes underwent a progressive maximal cycling ergometer test with concurrent automatic brachial BP measurement and ECG recording (Schiller AG^®^, Switzerland). The exercise test protocol started with a load level of 100 W after a 2-min warm-up period that was conducted with 50 W. Loads were increased by 50 W every 2 min until exhaustion, which was defined as the participant’s inability to maintain the load for 2 min. Next, the load was decreased to 25 W for 3 min of active recovery, followed by a 2-min cool-down period at rest. The test concluded with a final ECG recording and a brachial BP measurement. BP (systolic and diastolic) was measured once a minute during test and recovery periods, including at the maximum workload, immediately after the maximum workload, immediately after the end of the test, and after 5 min of recovery. Heart rate was measured with continuous ECG recording throughout the test and recovery periods. We assessed the absolute maximum power output (MPO) of the athletes as well as the MPO adjusted to individual body weight. Other measurements included maximum heart rate and heart rate at rest and 5 min after the exercise test.

Increases in systolic and diastolic BP were calculated from peak and baseline (resting) values and determined as blood pressure response (BPR). Further, the workload-adjusted BPR was calculated via maximum systolic BP/MPO. Pulse pressure was calculated as systolic—diastolic BP at rest and at maximum exercise conditions. In addition, mean BP was determined as: diastolic BP + (systolic BP-diastolic BP)/3.

### Statistical analysis

Descriptive analyses were carried out on all study variables for the total sample. Further, descriptive statistics were used on all study variables by 25-OH vitamin D status (with < 30 ng/mL classified as “insufficient” and ≥ 30 ng/mL regarded as “sufficient”). All data are presented as mean ± standard deviation (SD). The Shapiro–Wilk test was used to determine normal distribution. In case of skewed distribution of the data, all analyses were performed on normalized data. Between-group comparisons (insufficient vs. sufficient 25-OH vitamin D levels) were made using independent sample *t* tests. Bivariate relations were analysed using Pearson’s product-moment correlation coefficient. Statistical significance was set at *p* < 0.05 (two-tailed) for all measurements. All statistical analyses were performed using the statistical software SPSS 25.0 for Mac (Statistical Package for the Social Sciences, Chicago, IL, USA).

## Results

### Cohort characteristics

The 120 professional handball and ice hockey athletes (mean age of 25.8 ± 5.2 year) included in the study were experienced athletes and had participated in professional training for 9.6 ± 5 year with a current mean training time of 17.5 ± 3.0 h per week. The height was 189.9 ± 7.2 cm and the mean weight was 92.9 ± 10.6 kg, resulting in mean body mass index of 25.7 ± 1.85 kg/m^2^ (Table 1).

**Table 1:** Characteristics of all 120 professional indoor athletes

	Mean	SD
Age (years)	25.8	5.2
Height (cm)	189.8	7.2
Weight (kg)	92.9	10.6
Body mass index (kg/m^2^)	25.7	1.85
History of training (years)	9.6	5
Training per week (h)	17.5	3

The mean 25-OH vitamin D level of the 120 athletes was 37 ± 11.9 ng/mL. Eighty-five athletes (71%) displayed sufficient vitamin D levels of ≥ 30 ng/mL and 35 (29%) were found to be vitamin D insufficient (< 30 ng/mL). There were no significant between-group differences in age and characteristics. As expected, the 25-OH vitamin D levels between the sufficient and insufficient groups (42.3 ± 8.9 ng/mL vs. 22.95 ± 5.1 ng/mL) were different (*p* < 0.001). Table 2 shows the between-group comparison for all parameters measured.

**Table 2:** Comparison of the different characteristics according to 25-OH vitamin D levels

	25-OH vitamin D levels				*p* value
	≥ 30 ng/mL		< 30 ng/mL		
	*n* = 85		*n* = 35		
	Mean	SD	Mean	SD	
25-OH vitamin D (ng/mL)	42.3	8.9	22.95	5.1	**< 0.001**
Age (years)	25.5	4.8	27.5	5.8	0.063
Weight (kg)	92.5	10.5	94	11	0.505
Height (cm)	189.6	7.0	190	8.1	0.661
BMI (kg/m^2^)	25.7	1.9	25.9	1.5	0.611
Body surface area (m^2^)	2.2	0.15	2.22	0.17	0.532
Heart rate at rest (bpm)	58.4	10.5	59.1	9.4	0.748
Brachial systolic blood pressure (mmHg)	122.1	10	120	11.6	0.333
Brachial diastolic blood pressure (mmHg)	75.1	7.4	76.8	8.6	0.362
Mean brachial blood pressure (mmHg)	91	6.3	90.7	6.5	0.862
Brachial pulse pressure (mmHg)	47	11	43.5	13.3	0.152
Maximum heart rate (bpm)	184	8.4	183.7	7.2	0.862
Maximum systolic brachial blood pressure (mmHg)	197.8	17.5	189.3	19.4	**0.026**
Mean maximum brachial blood pressure (mmHg)	116.7	8	119.1	8.2	0.169
Increase from resting systolic blood pressure (mmHg)	75.7	20	69.3	21.6	0.103
Brachial pulse pressure exercise (mmHg)	118	18	108.9	20.8	**0.021**
Maximum diastolic brachial blood pressure (mmHg)	79.7	7.4	80.4	7.3	0.651
Increase from resting diastolic blood pressure (mmHg)	4.6	5.7	3.4	6.3	0.621
Maximum work load (W)	367.2	78.4	332.8	79.9	**0.039**
Maximum power output (MPO) (W/kg)	3.99	0.82	3.58	0.78	**0.015**
Max. systolic blood pressure/MPO (mmHg × kg/W)	51.3	10.2	55.5	14.1	0.079
Parathyroid hormone (pg/mL)	31.2	17.5	42.3	17.5	**0.003**
Calcium (mmol/L)	2.38	0.09	2.34	0.14	0.142
Magnesium (mmol/L)	0.82	0.05	0.82	0.06	0.713
Hemoglobin (g/dL)	14.9	1	15.1	0.85	0.402
Hematocrit (Vol%)	42.7	2.4	42.9	2.2	0.699

Bold text signifies significant differences. Values are given as means ± standard deviation (SD)

### 25-OH vitamin D and resting blood pressure

The mean systolic/diastolic BP in all 120 athletes was 121 ± 10/75 ± 7 mmHg. The level of 25-OH vitamin D was positively correlated with systolic BP (*r*^2^ = 0.186, *p* = 0.045), whereas 25-OH vitamin D level and diastolic BP were not correlated (*r*^2^ = − 0.10, *p* = 0.28). Further, there was a positive correlation of 25-OH vitamin D level and pulse pressure at rest (*r*^2^ = 0.209, *p* = 0.024). None of the other measured BP parameters correlated with the 25-OH vitamin D status (Table 3).

**Table 3:** Spearman correlation coefficients for each variable for 120 athletes

Variable	Correlation with 25-OH Vitamin D	*p* value
	Concentration	
Age	− 0.072	0.439
Weight	− 0.011	0.90
Height	− 0.052	0.577
BMI	0.049	0.594
History of training	− 0.032	0.733
Training per week	0.049	0.599
Resting heart rate	0.012	0.905
Systolic blood pressure	0.186	**0.045**
Diastolic blood pressure	− 0.101	0.280
Pulse pressure at rest	0.209	**0.024**
Mean brachial blood pressure	0.014	0.881
Maximum heart rate	0.081	0.387
Maximum systolic blood pressure	0.078	0.401
Maximum diastolic blood pressure	0.010	0.915
Pulse pressure exercise	0.095	0.308
Mean blood pressure exercise	0.071	0.527
Increase from resting systolic BP	− 0.026	0.777
Increase from resting diastolic BP	0.113	0.223
Increase from resting mean BP	0.059	0.527
Maximum workload	0.373	**< 0.001**
Maximum power output (MPO)	0.327	**< 0.001**
Maximum systolic BP/ MPO	− 0.222	**0.015**
Parathyroid hormone	− 0.330	**< 0.001**
Calcium	0.229	**0.013**
Magnesium	− 0.077	0.422
Hemoglobin	0.067	0.477
Hematocrit	0.014	0.877

Bold text signifies a significant correlation

There were no differences in resting systolic and diastolic BP, pulse pressure, and mean BP between athletes with insufficient and sufficient 25-OH vitamin D levels. Detailed data are given in Table 2.

### 25-OH vitamin D and blood pressure response during maximum exercise

The mean maximum BP in all athletes was 193 ± 21/80 ± 7 mmHg, with a mean systolic BP increase of 72 ± 22 mmHg and a mean diastolic BP increase of 4 ± 6 mmHg. The maximum pulse pressure was 113 ± 18 mmHg.

Athletes with sufficient 25-OH vitamin D levels had a higher maximum systolic BP compared with those with insufficient levels (198 ± 17 vs. 189 ± 19, *p* = 0.026). In addition, the maximum pulse pressure (118 ± 18 vs. 109 ± 21 mmHg, *p* = 0.021) was higher in athletes with sufficient 25-OH vitamin D levels, whereas the individual BPR was not different between groups (76 ± 20/5 ± 6 vs. 69 ± 22/3 ± 6 mmHg, *p* = 0.103). Detailed data are given in Table 2. In the total sample, 25-OH vitamin D levels did not correlate with any of the exercise-induced changes in BP (Table 3).

### 25-OH vitamin D and performance

All athletes completed the maximum exercise test until exhaustion and a maximum heart rate above the calculated individual 85% threshold (of individually calculated maximum heart rate). The mean maximum heart rate in all athletes was 184 ± 8 bpm and the mean workload was 349 ± 85 W with a corresponding maximum power output (MPO) of 3.77 ± 0.85 W/kg. The workload-adjusted BPR, calculated via maximum systolic BP/MPO, was 53.5 ± 12 mmHg × kg/W. Levels of 25-OH vitamin D correlated positively with maximum workload (*r*^2^ = 0.373, *p* < 0.001) as well as maximum power output (*r*^2^ = 0.327, *p* < 0.001), whereas 25-OH vitamin D levels and the workload-adjusted maximum systolic BP (*r*^2^ = − 0.222, *p* = 0.015) were negatively correlated. All other exercise-induced BP measurements did not correlate with 25-OH vitamin D levels (Table 3).

As shown in Table 2, athletes with sufficient vitamin D levels had a higher maximum power output and achieved higher workloads than those with insufficient levels. The workload-adjusted BPR was not different between vitamin D-sufficient and -insufficient athletes. Maximum heart rate was also not different between the groups.

Linear regression analyses revealed that a higher level of 25- vitamin D (*p* < 0.001) is a statistically significant predictor of a higher maximum output (*r*^2^ = 0.123, corrected *r*^2^ = 0.115, *p* < 0.001, F(1120) = 16, Durbin-Watson statistic 2.123) and of a higher maximum workload (*r*^2^ = 0.126, corrected *r*^2^ = 0.118, *p* < 0.001, F(1120) = 16.5, Durbin-Watson statistic 2.065). Thus, 25- OH vitamin D levels were not able to predict the workload-adjusted BPR (*r*^2^ = 0.078, corrected *r*^2^ = 0.070, *p* = 0.12, F(1120) = 92.3, Durbin-Watson statistic 1.23).

### 25-OH vitamin D and blood parameters

Mean parathyroid hormone levels were 34 ± 18 ng/mL, calcium levels were 2.37 ± 0.11 mmol/L, and magnesium levels were 0.82 ± 0.05 mmol/L for all athletes. Mean haemoglobin levels were 15 ± 0.89 g/dL and the mean haematocrit was 42.8 ± 2.24%. We found a negative correlation of 25-OH vitamin D with parathyroid hormone (*r*^2^ = − 0.330, *p* < 0.001) and a positive correlation with calcium levels (*r*^2^ = 0.229, *p* = 0.013). The other parameters tested did not correlate with the 25-OH vitamin D status (Table 3).

Athletes with insufficient 25-OH vitamin D levels displayed higher parathyroid hormone levels compared with athletes with sufficient vitamin D (42.3 ± 17.5 vs. 31.2 ± 17.5 ng/mL, *p* = 0.003). The other measured blood parameters did not show differences between the two groups (Table 2).

## Discussion

To the best of our knowledge, our study is the first to investigate the blood pressure response to a standardized maximum exercise test in professional indoor athletes in Germany and its correlation with 25-OH vitamin D status. Our main findings are that athletes with sufficient vitamin D levels displayed a higher maximum systolic BP and maximum pulse pressure during exercise compared with athletes with insufficient vitamin D levels. Further, the achieved maximum workload and MPO were higher in athletes with sufficient vitamin D levels. The BP increases during exercise (BPR) and the workload-adjusted BPR were not different between the sufficient and insufficient groups, however, indicating that the higher maximum systolic BP in sufficient athletes was caused by the better MPO. This assumption is underpinned by the lack of a correlation of vitamin D levels with both the maximum systolic BP and the BPR. Further, 25-OH vitamin D levels predicted maximum workload and MPO, but not the workload-indexed BPR.

These findings were surprising, since we hypothesized that athletes with insufficient vitamin D levels would display both a higher BP at rest and a higher BPR to a maximum exercise test compared with athletes with sufficient vitamin D levels. These assumptions were based on our own recently published research results in which we demonstrated that vitamin D-insufficient elite handball athletes displayed a higher peripheral and central BP than those with sufficient vitamin D levels (Bauer et al. 2019). Thus, contrary to our hypothesis, the current study showed that vitamin D levels were not associated with the BPR and the maximum BP during a maximum exercise. Athletes with sufficient vitamin D levels even had a higher maximum systolic BP than insufficient athletes, which was explained by the better performance of vitamin D-sufficient athletes.

Interestingly, the detected maximum systolic BP of our professional indoor athletes (193 ± 21 mmHg) was lower than that reported by Pressler et al. (Pressler et al. 2018), who found a mean maximum BP of 204 ± 22 mmHg in their male professional athletes. Accordingly, the mean increase from resting systolic BP was lower in our cohort than in theirs (72 ± 21 vs. 80 ± 20 mmHg), and the maximum diastolic BP was slightly higher in our cohort (80 ± 7 vs. 77 ± 9 mmHg) both in the total cohort and in the vitamin D-sufficient group. The study of Pressler at el. seems to be the most appropriate study to compare with ours, since their male cohort was of similar age and displayed a similar maximum heart rate (our study 184 ± 8 vs. Pressler et al. 186 ± 12 bpm) and a comparable MPO (3.77 ± 0.85 vs. 4.15 ± 0.61 W/kg). In addition, resting heart rate (59 ± 10 vs. 60 ± 11 bpm) and resting BP (122 ± 10/75 ± 7 vs. 124 ± 12/77 ± 7 mmHg) were comparable in the two study populations. Unfortunately, the lack of assessment of vitamin D levels in the report by Pressler et al. (Pressler et al. 2018) limits the comparison with our own results.

Another current study by Caselli et al. addressing the BPR to a maximum exercise test in Olympic athletes reported a maximum BP of 190 ± 20/75 ± 8 mmHg (Caselli et al. 2016), which is lower than the value we measured. Athletes performing “mixed exercise” demonstrated the highest resting BP in this study (Caselli et al. 2016), which is in line with other publications (Berge et al. 2015; Pressler et al. 2018; Hedman et al. 2019b). Further, male athletes have been shown to display a higher resting BP than females (Hedman et al. 2019b; Pressler et al. 2018; Berge et al. 2015). Our cohort, consisting of male handball and ice hockey players can also be considered “mixed exercise” athletes. Unfortunately, the data presented by Caselli et al. for mixed sports are not divided by gender, which limits comparison. The maximum workload in this study (Caselli et al. 2016) was significantly lower compared to that of our group (268 ± 53 vs. 349 ± 85 W) and, in addition, the maximum heart rate was significantly lower (166 ± 10 vs. 184 ± 8 bpm). The mean age of 25 ± 6 years and the surprisingly low maximum heart rate raises the question whether the accomplished exercise test can be really considered to be a maximum exercise test. Further, lack of assessment of 25-OH vitamin D precludes correlation with vitamin D status and thus limits the comparison with our results.

Only a few studies have addressed the association of 25-OH vitamin D and BPR with exercise (Zaleski et al. 2019; Babur Guler et al. 2016). None, to the best of our knowledge, have examined professional athletes, a population that has been repeatedly reported to be vitamin D insufficient (Bauer et al. 2018; Owens et al. 2018, 2015). In 417 healthy adults (mean age 44), Zaleski et al. (Zaleski et al. 2019) reported that they found no difference in the BPR to a maximal exercise test and in the maximum BP between probands with insufficient and those with sufficient 25-OH vitamin D concentrations. Only 52% of the participants displayed normal resting BP, however, and 22.5% were considered to have established hypertension. Further, the cohort age was given as 20–76 years, data were not stratified by sex, and > 50% of the participants were females, who display a lower BPR than males (Zaleski et al. 2019). Therefore, these results cannot be meaningfully compared with the findings of our study. This previous study, however, was the first to examine the association of vitamin D levels with the BPR to exercise in a healthy population. Interestingly, the reported maximum BP in this investigation (Zaleski et al. 2019) was considerably lower than in our study and the studies of Caselli et al. (Caselli et al. 2016) and Pressler et al. (Pressler et al. 2018). This finding might be explained by the lower performance of the participants (VO2 max. 34 mL/kg × min), as a normal, healthy population was examined instead of athletes (Zaleski et al. 2019).

In our study, we identified a highly significant correlation of vitamin D levels and MPO. The influence of vitamin D on physical performance levels of male professional athletes has been described in several studies (Hamilton et al. 2014; Ksiazek et al. 2016), which can be explained via both its musculoskeletal and cardiovascular effects (Cannell et al. 2009; Owens et al. 2015; Wang 2016; Allison et al. 2015); however, the influence of vitamin D on the BPR to exercise was not examined in athletes thus far. The higher maximum BP that we measured in athletes with sufficient 25-OH vitamin D levels might be explained by a higher performance. It is known that higher exercise performance correlates with a higher BPR and higher maximum BP in athletes. Therefore, the workload adjustment in the BPR to exercise was proposed to identify athletes with an “exaggerated” BPR instead of using absolute cut-off values (Hedman et al. 2019a). Several mechanisms have been discussed to contribute to the excessive increase in BP during exercise, including aortic distensibility (Roman et al. 2007; Thijssen et al. 2016), endothelial dysfunction (Tzemos et al. 2009), and increased activation of the renin–angiotensin–aldosterone system (Schultz et al. 2013). Interestingly, all of these modifiers of the BPR to exercise are influenced by 25-OH vitamin D (Norman 2008; Tarcin et al. 2009; Christakos et al. 2013).

We could not detect correlations of vitamin D levels with BP changes under exercise conditions. This might be explained by the excellent fitness of our professional athletes, since it is possible that vitamin D and physical fitness differentially affect the cardiovascular system and the BPR to exercise, with measurable differences only in individuals who cannot compensate for the deleterious effects of vitamin D insufficiency (Norman 2008; Tzemos et al. 2009). Today, the ability to examine vascular function and central haemodynamic parameters including central BP (cBP) via non-invasive tools might offer new opportunities to reveal the influence of 25-OH vitamin D on BP and the BPR to exercise in an athletic population (Bauer et al. 2019). cBP is determined by the complex interaction between aortic compliance, elasticity and the resistance arteries’ ability to channel blood flow in accordance with tissue needs (Stephen Hedley and Phelan 2017). Hence, cBP is superior to brachial blood pressure in identifying changes in vascular functional parameters (Hodson et al. 2016) and cardiovascular risk (Williams et al. 2006; Roman et al. 2007; Cheng et al. 2013; Fan et al. 2016; Herbert et al. 2014). Particularly in young men, central diastolic BP, but not peripheral systolic pressure, is a predictor of cardiovascular risk (Wilkinson et al. 2001). Therefore, further studies that investigate the impact of 25-OH vitamin D on vascular function, cBP, and the BPR to exercise in athletes are needed.

Overall, the clinical importance of the BPR to exercise and maximum systolic BP is obvious, as it has been shown that a high BPR to exercise predicts future development of hypertension in young athletes (Caselli et al. 2019) as well as the normal population (Tzemos et al. 2015; Percuku et al. 2019; Schultz et al. 2015). Athletes are more frequently exposed to exercise-induced high BP than sedentary individuals, and exercise testing is frequently performed in the cardiovascular evaluation of competitive athletes (Hedman et al. 2019b). Therefore, reliable cut-off values have to be established and, given the workload-dependent BPR to exercise, a workload-adjusted approach instead of using absolute cut-off values might be a good strategy for identifying athletes with an exaggerated BPR (Hedman et al. 2019a). Thus far, insufficient 25-OH vitamin D levels do not appear to be a risk factor for an exaggerated BPR to a maximum exercise test in professional male indoor athletes.

### Limitations

Our study has a few noteworthy limitations. The number of participants limited its power to uncover potential correlations between 25-OH vitamin D status and markers of cardiac and vascular function other than brachial BP and the BPR to a maximum exercise test. The focus on professional indoor handball and ice hockey players may limit extrapolation of the results to other sport disciplines; however, as these team sports expose athletes to the haemodynamic stress of frequent interval sprints, they are representative of other sports with a high dynamic component (> 75% VO2max) and a moderate static component (10–20%) (Levine et al. 2015). A further limitation is our exclusive focus on male athletes, which precludes the extrapolation of our results to female athletes. We are currently addressing all three issues by extending our research to include larger numbers of professional male and female athletes from various sports disciplines.

## Conclusion

We have shown for the first time that professional male handball and ice hockey players with sufficient 25-OH vitamin D levels display a higher maximum systolic BP during a maximum exercise test than do athletes with insufficient 25-OH vitamin D levels. This difference was an expression of a significantly higher MPO in vitamin D-sufficient athletes, highlighting the impact of vitamin D on physical performance of these athletes. Thus, the BPR between athletes with sufficient 25- OH vitamin D levels compared to those with insufficient levels was not different. Further, in both groups of athletes, the BPR to a maximum exercise did not exceed the currently proposed thresholds for athletes.

We suggest that highly- trained athletic populations present a unique opportunity to address the modifying effects of cardiorespiratory fitness on the association of 25-OH vitamin D with cardiovascular risk markers. Our results may serve to stimulate future investigations into the correlation of vitamin D with parameters of vascular function and central haemodynamic parameters.

## Abbreviations

BP: Blood pressure
BPR: Blood pressure response
cBP: Central blood pressure
ECG: Electrocardiogram
K2EDTA: Dipotassium ethylenediamine tetra-acetic acid
MPO: Maximum power output
SD: Standard deviation
VO2max: Maximum oxygen uptake

## References

[CR1] Al Mheid I, Patel R, Murrow J, Morris A, Rahman A, Fike L, Kavtaradze N, Uphoff I, Hooper C, Tangpricha V, Alexander RW, Brigham K, Quyyumi AA (2011). Vitamin D status is associated with arterial stiffness and vascular dysfunction in healthy humans. J Am Coll Cardiol.

[CR2] Allison RJ, Close GL, Farooq A, Riding NR, Salah O, Hamilton B, Wilson MG (2015). Severely vitamin D-deficient athletes present smaller hearts than sufficient athletes. Eur J Prev Cardiol.

[CR3] Anderson JL, May HT, Horne BD, Bair TL, Hall NL, Carlquist JF, Lappe DL, Muhlestein JB, Intermountain Heart Collaborative Study G (2010). Relation of vitamin D deficiency to cardiovascular risk factors, disease status, and incident events in a general healthcare population. Am J Cardiol.

[CR4] Babur Guler G, Guler E, Hatipoglu S, Gunes HM, Gecmen C, Demir GG, Barutcu I (2016). Assessment of 25-OH vitamin D levels and abnormal blood pressure response in female patients with cardiac syndrome X. Anatol J Cardiol.

[CR5] Bauer P, Henni S, Dorr O, Bauer T, Hamm CW, Most A (2018). High prevalence of vitamin D insufficiency in professional handball athletes. Phys Sportsmed.

[CR6] Bauer P, Kraushaar L, Holscher S, Tajmiri-Gondai S, Dorr O, Nef H, Hamm C, Most A (2019). Elite athletes as research model: vitamin D insufficiency associates with elevated central blood pressure in professional handball athletes. Eur J Appl Physiol.

[CR7] Berge HM, Isern CB, Berge E (2015). Blood pressure and hypertension in athletes: a systematic review. Br J Sports Med.

[CR8] Cannell JJ, Hollis BW, Sorenson MB, Taft TN, Anderson JJ (2009). Athletic performance and vitamin D. Med Sci Sports Exerc.

[CR9] Caselli S, Serdoz A, Mango F, Lemme E, Vaquer Segui A, Milan A, Attenhofer Jost C, Schmied C, Spataro A, Pelliccia A (2019). High blood pressure response to exercise predicts future development of hypertension in young athletes. Eur Heart J.

[CR10] Caselli S, Vaquer Segui A, Quattrini F, Di Gacinto B, Milan A, Assorgi R, Verdile L, Spataro A, Pelliccia A (2016). Upper normal values of blood pressure response to exercise in Olympic athletes. Am Heart J.

[CR11] Cheng HM, Chuang SY, Sung SH, Yu WC, Pearson A, Lakatta EG, Pan WH, Chen CH (2013). Derivation and validation of diagnostic thresholds for central blood pressure measurements based on long-term cardiovascular risks. J Am Coll Cardiol.

[CR12] Christakos S, Hewison M, Gardner DG, Wagner CL, Sergeev IN, Rutten E, Pittas AG, Boland R, Ferrucci L, Bikle DD (2013). Vitamin D: beyond bone. Ann N Y Acad Sci.

[CR13] Currie KD, Floras JS, La Gerche A, Goodman JM (2018). Exercise blood pressure guidelines: time to re-evaluate what is normal and exaggerated?. Sports Med.

[CR14] Du Bois D, Du Bois EF (1989). A formula to estimate the approximate surface area if height and weight be known 1916. Nutrition.

[CR15] Fan F, Qi L, Jia J, Xu X, Liu Y, Yang Y, Qin X, Li J, Li H, Zhang Y, Huo Y (2016). Noninvasive central systolic blood pressure is more strongly related to kidney function decline than peripheral systolic blood pressure in a Chinese community-based population. Hypertension.

[CR16] Forman JP, Williams JS, Fisher ND (2010). Plasma 25-hydroxyvitamin D and regulation of the renin-angiotensin system in humans. Hypertension.

[CR17] Gupta MP, Polena S, Coplan N, Panagopoulos G, Dhingra C, Myers J, Froelicher V (2007). Prognostic significance of systolic blood pressure increases in men during exercise stress testing. Am J Cardiol.

[CR18] Hamilton B, Whiteley R, Farooq A, Chalabi H (2014). Vitamin D concentration in 342 professional football players and association with lower limb isokinetic function. J Sci Med Sport.

[CR19] Harriss DJ, Macsween A, Atkinson G (2017). Standards for ethics in sport and exercise science research: 2018 update. Int J Sports Med.

[CR20] He CS, Aw Yong XH, Walsh NP, Gleeson M (2016). Is there an optimal vitamin D status for immunity in athletes and military personnel?. Exerc Immunol Rev.

[CR21] Hedman K, Cauwenberghs N, Christle JW, Kuznetsova T, Haddad F, Myers J (2019). Workload-indexed blood pressure response is superior to peak systolic blood pressure in predicting all-cause mortality. Eur J Prev Cardiol.

[CR22] Hedman K, Moneghetti KJ, Christle JW, Bagherzadeh SP, Amsallem M, Ashley E, Froelicher V, Haddad F (2019). Blood pressure in athletic preparticipation evaluation and the implication for cardiac remodelling. Heart.

[CR23] Herbert A, Cruickshank JK, Laurent S, Boutouyrie P, Reference Values for Arterial Measurements C (2014). Establishing reference values for central blood pressure and its amplification in a general healthy population and according to cardiovascular risk factors. Eur Heart J.

[CR24] Hodson B, Norton GR, Booysen HL, Sibiya MJ, Raymond A, Maseko MJ, Majane OH, Libhaber E, Sareli P, Woodiwiss AJ (2016). Brachial pressure control fails to account for most distending pressure-independent, age-related aortic hemodynamic changes in adults. Am J Hypertens.

[CR25] Holick MF, Binkley NC, Bischoff-Ferrari HA, Gordon CM, Hanley DA, Heaney RP, Murad MH, Weaver CM (2012). Guidelines for preventing and treating vitamin D deficiency and insufficiency revisited. J Clin Endocrinol Metab.

[CR26] Holick MF, Binkley NC, Bischoff-Ferrari HA, Gordon CM, Hanley DA, Heaney RP, Murad MH, Weaver CM, Endocrine S (2011). Evaluation, treatment, and prevention of vitamin D deficiency: an endocrine society clinical practice guideline. J Clin Endocrinol Metab.

[CR27] Holmqvist L, Mortensen L, Kanckos C, Ljungman C, Mehlig K, Manhem K (2012). Exercise blood pressure and the risk of future hypertension. J Hum Hypertens.

[CR28] Kim D, Ha JW (2016). Hypertensive response to exercise: mechanisms and clinical implication. Clin Hypertens.

[CR29] Krzywanski J, Mikulski T, Krysztofiak H, Mlynczak M, Gaczynska E, Ziemba A (2016). Seasonal vitamin D status in Polish elite athletes in relation to sun exposure and oral supplementation. PLoS ONE.

[CR30] Ksiazek A, Zagrodna A, Dziubek W, Pietraszewski B, Ochmann B, Slowinska-Lisowska M (2016). 25(OH)D3 levels relative to muscle strength and maximum oxygen uptake in athletes. J Hum Kinet.

[CR31] Kunutsor SK, Apekey TA, Steur M (2013). Vitamin D and risk of future hypertension: meta-analysis of 283,537 participants. Eur J Epidemiol.

[CR32] Levine BD, Baggish AL, Kovacs RJ, Link MS, Maron MS, Mitchell JH, American Heart Association E, Arrhythmias Committee of Council on Clinical Cardiology CoCDiYCoC, Stroke Nursing CoFG, Translational B, American College of C (2015). Eligibility and disqualification recommendations for competitive athletes with cardiovascular abnormalities: task force 1: classification of sports: dynamic, static, and impact: a scientific statement from the American heart association and American college of cardiology. Circulation.

[CR33] Lewis GD, Gona P, Larson MG, Plehn JF, Benjamin EJ, O'Donnell CJ, Levy D, Vasan RS, Wang TJ (2008). Exercise blood pressure and the risk of incident cardiovascular disease (from the Framingham Heart Study). Am J Cardiol.

[CR34] Mancia G, Fagard R, Narkiewicz K, Redon J, Zanchetti A, Bohm M, Christiaens T, Cifkova R, De Backer G, Dominiczak A, Galderisi M, Grobbee DE, Jaarsma T, Kirchhof P, Kjeldsen SE, Laurent S, Manolis AJ, Nilsson PM, Ruilope LM, Schmieder RE, Sirnes PA, Sleight P, Viigimaa M, Waeber B, Zannad F, Redon J, Dominiczak A, Narkiewicz K, Nilsson PM, Burnier M, Viigimaa M, Ambrosioni E, Caufield M, Coca A, Olsen MH, Schmieder RE, Tsioufis C, van de Borne P, Zamorano JL, Achenbach S, Baumgartner H, Bax JJ, Bueno H, Dean V, Deaton C, Erol C, Fagard R, Ferrari R, Hasdai D, Hoes AW, Kirchhof P, Knuuti J, Kolh P, Lancellotti P, Linhart A, Nihoyannopoulos P, Piepoli MF, Ponikowski P, Sirnes PA, Tamargo JL, Tendera M, Torbicki A, Wijns W, Windecker S, Clement DL, Coca A, Gillebert TC, Tendera M, Rosei EA, Ambrosioni E, Anker SD, Bauersachs J, Hitij JB, Caulfield M, De Buyzere M, De Geest S, Derumeaux GA, Erdine S, Farsang C, Funck-Brentano C, Gerc V, Germano G, Gielen S, Haller H, Hoes AW, Jordan J, Kahan T, Komajda M, Lovic D, Mahrholdt H, Olsen MH, Ostergren J, Parati G, Perk J, Polonia J, Popescu BA, Reiner Z, Ryden L, Sirenko Y, Stanton A, Struijker-Boudier H, Tsioufis C, van de Borne P, Vlachopoulos C, Volpe M, Wood DA (2013). 2013 ESH/ESC guidelines for the management of arterial hypertension: the task force for the management of arterial hypertension of the European society of hypertension (ESH) and of the European society of cardiology (ESC). Eur Heart J.

[CR35] Maroon JC, Mathyssek CM, Bost JW, Amos A, Winkelman R, Yates AP, Duca MA, Norwig JA (2015). Vitamin D profile in national football league players. Am J Sports Med.

[CR36] Mehran N, Schulz BM, Neri BR, Robertson WJ, Limpisvasti O (2016). Prevalence of vitamin D insufficiency in professional hockey players. Orthop J Sports Med.

[CR37] Morton JP, Iqbal Z, Drust B, Burgess D, Close GL, Brukner PD (2012). Seasonal variation in vitamin D status in professional soccer players of the english premier league. Appl Physiol Nutr Metab.

[CR38] Norman AW (2008). From vitamin D to hormone D: fundamentals of the vitamin D endocrine system essential for good health. Am J Clin Nutr.

[CR39] Owens DJ, Allison R, Close GL (2018). Vitamin D and the athlete: current perspectives and new challenges. Sports Med.

[CR40] Owens DJ, Fraser WD, Close GL (2015). Vitamin D and the athlete: emerging insights. Eur J Sport Sci.

[CR41] Percuku L, Bajraktari G, Jashari H, Bytyci I, Ibrahimi P, Henein MY (2019). The exaggerated systolic hypertensive response to exercise associates cardiovascular events: a systematic review and meta-analysis. Pol Arch Intern Med.

[CR42] Pludowski P, Karczmarewicz E, Bayer M, Carter G, Chlebna-Sokol D, Czech-Kowalska J, Debski R, Decsi T, Dobrzanska A, Franek E, Gluszko P, Grant WB, Holick MF, Yankovskaya L, Konstantynowicz J, Ksiazyk JB, Ksiezopolska-Orlowska K, Lewinski A, Litwin M, Lohner S, Lorenc RS, Lukaszkiewicz J, Marcinowska-Suchowierska E, Milewicz A, Misiorowski W, Nowicki M, Povoroznyuk V, Rozentryt P, Rudenka E, Shoenfeld Y, Socha P, Solnica B, Szalecki M, Talalaj M, Varbiro S, Zmijewski MA (2013). Practical guidelines for the supplementation of vitamin D and the treatment of deficits in Central Europe—recommended vitamin D intakes in the general population and groups at risk of vitamin D deficiency. Endokrynol Pol.

[CR43] Pressler A, Jahnig A, Halle M, Haller B (2018). Blood pressure response to maximal dynamic exercise testing in an athletic population. J Hypertens.

[CR44] Priemel M, von Domarus C, Klatte TO, Kessler S, Schlie J, Meier S, Proksch N, Pastor F, Netter C, Streichert T, Puschel K, Amling M (2010). Bone mineralization defects and vitamin D deficiency: histomorphometric analysis of iliac crest bone biopsies and circulating 25-hydroxyvitamin D in 675 patients. J Bone Miner Res.

[CR45] Roman MJ, Devereux RB, Kizer JR, Lee ET, Galloway JM, Ali T, Umans JG, Howard BV (2007). Central pressure more strongly relates to vascular disease and outcome than does brachial pressure: the strong heart study. Hypertension.

[CR46] Schultz MG, Otahal P, Cleland VJ, Blizzard L, Marwick TH, Sharman JE (2013). Exercise-induced hypertension, cardiovascular events, and mortality in patients undergoing exercise stress testing: a systematic review and meta-analysis. Am J Hypertens.

[CR47] Schultz MG, Otahal P, Picone DS, Sharman JE (2015). Clinical relevance of exaggerated exercise blood pressure. J Am Coll Cardiol.

[CR48] Somjen D, Weisman Y, Kohen F, Gayer B, Limor R, Sharon O, Jaccard N, Knoll E, Stern N (2005). 25-hydroxyvitamin D3–1alpha-hydroxylase is expressed in human vascular smooth muscle cells and is upregulated by parathyroid hormone and estrogenic compounds. Circulation.

[CR49] Stephen Hedley J, Phelan D (2017). Athletes and the aorta: normal adaptations and the diagnosis and management of pathology. Curr Treat Options Cardiovasc Med.

[CR50] Tahir E, Starekova J, Muellerleile K, von Stritzky A, Munch J, Avanesov M, Weinrich JM, Stehning C, Bohnen S, Radunski UK, Freiwald E, Blankenberg S, Adam G, Pressler A, Patten M, Lund GK (2018). Myocardial fibrosis in competitive triathletes detected by contrast-enhanced CMR correlates with exercise-induced hypertension and competition history. JACC Cardiovasc Imaging.

[CR51] Tarcin O, Yavuz DG, Ozben B, Telli A, Ogunc AV, Yuksel M, Toprak A, Yazici D, Sancak S, Deyneli O, Akalin S (2009). Effect of vitamin D deficiency and replacement on endothelial function in asymptomatic subjects. J Clin Endocrinol Metab.

[CR52] Thijssen DH, Carter SE, Green DJ (2016). Arterial structure and function in vascular ageing: are you as old as your arteries?. J Physiol.

[CR53] Tzemos N, Lim PO, MacDonald TM (2009). Exercise blood pressure and endothelial dysfunction in hypertension. Int J Clin Pract.

[CR54] Tzemos N, Lim PO, Mackenzie IS, MacDonald TM (2015). Exaggerated exercise blood pressure response and future cardiovascular disease. J Clin Hypertens (Greenwich).

[CR55] Vimaleswaran KS, Cavadino A, Berry DJ, Jorde R, Dieffenbach AK, Lu C, Alves AC, Heerspink HJ, Tikkanen E, Eriksson J, Wong A, Mangino M, Jablonski KA, Nolte IM, Houston DK, Ahluwalia TS, van der Most PJ, Pasko D, Zgaga L, Thiering E, Vitart V, Fraser RM, Huffman JE, de Boer RA, Schottker B, Saum KU, McCarthy MI, Dupuis J, Herzig KH, Sebert S, Pouta A, Laitinen J, Kleber ME, Navis G, Lorentzon M, Jameson K, Arden N, Cooper JA, Acharya J, Hardy R, Raitakari O, Ripatti S, Billings LK, Lahti J, Osmond C, Penninx BW, Rejnmark L, Lohman KK, Paternoster L, Stolk RP, Hernandez DG, Byberg L, Hagstrom E, Melhus H, Ingelsson E, Mellstrom D, Ljunggren O, Tzoulaki I, McLachlan S, Theodoratou E, Tiesler CM, Jula A, Navarro P, Wright AF, Polasek O, Caroline H, Wilson JF, Rudan I, Salomaa V, Heinrich J, Campbell H, Price JF, Karlsson M, Lind L, Michaelsson K, Bandinelli S, Frayling TM, Hartman CA, Sorensen TI, Kritchevsky SB, Langdahl BL, Eriksson JG, Florez JC, Spector TD, Lehtimaki T, Kuh D, Humphries SE, Cooper C, Ohlsson C, Marz W, de Borst MH, Kumari M, Kivimaki M, Wang TJ, Power C, Brenner H, Grimnes G, van der Harst P, Snieder H, Hingorani AD, Pilz S, Whittaker JC, Jarvelin MR, Hypponen E, LifeLines Cohort Study i, International Consortium for Blood P, Cohorts for H, Aging Research in Genomic Epidemiology c, Global Blood Pressure Genetics c (2014). Association of vitamin D status with arterial blood pressure and hypertension risk: a mendelian randomisation study. Lancet Diabetes Endocrinol.

[CR56] Wang TJ (2016). Vitamin D and cardiovascular disease. Annu Rev Med.

[CR57] Weiss SA, Blumenthal RS, Sharrett AR, Redberg RF, Mora S (2010). Exercise blood pressure and future cardiovascular death in asymptomatic individuals. Circulation.

[CR58] Wilkinson IB, Franklin SS, Hall IR, Tyrrell S, Cockcroft JR (2001). Pressure amplification explains why pulse pressure is unrelated to risk in young subjects. Hypertension.

[CR59] Williams B, Lacy PS, Thom SM, Cruickshank K, Stanton A, Collier D, Hughes AD, Thurston H, O'Rourke M, Investigators C, Anglo-Scandinavian Cardiac Outcomes Trial I, Committee CS, Writing C (2006). Differential impact of blood pressure-lowering drugs on central aortic pressure and clinical outcomes: principal results of the conduit artery function evaluation (CAFE) study. Circulation.

[CR60] Williams B, Mancia G, Spiering W, Agabiti Rosei E, Azizi M, Burnier M, Clement DL, Coca A, de Simone G, Dominiczak A, Kahan T, Mahfoud F, Redon J, Ruilope L, Zanchetti A, Kerins M, Kjeldsen SE, Kreutz R, Laurent S, Lip GYH, McManus R, Narkiewicz K, Ruschitzka F, Schmieder RE, Shlyakhto E, Tsioufis C, Aboyans V, Desormais I, Group ESCSD (2018). 2018 ESC/ESH guidelines for the management of arterial hypertension. Eur Heart J.

[CR61] Zaleski A, Taylor B, Armstrong B, Puglisi M, Clarkson P, Chipkin S, White CM, Thompson PD, Pescatello LS (2019). Associations of 25-hydroxyvitamin D With the blood pressure response to maximal exercise among healthy adults. Int J Sport Nutr Exerc Metab.

[CR62] Zorzi A, Perazzolo Marra M, Rigato I, De Lazzari M, Susana A, Niero A, Pilichou K, Migliore F, Rizzo S, Giorgi B, De Conti G, Sarto P, Serratosa L, Patrizi G, De Maria E, Pelliccia A, Basso C, Schiavon M, Bauce B, Iliceto S, Thiene G, Corrado D (2016). Nonischemic left ventricular scar as a substrate of life-threatening ventricular arrhythmias and sudden cardiac death in competitive athletes. Circ Arrhythm Electrophysiol.

